# Supplementary material for: Empirical methods for controlling false positives and estimating confidence in ChIP-Seq peaks
Source: BMC Bioinformatics. 2008 Dec 5;9:523. doi: 10.1186/1471-2105-9-523 (PMC2628906; doi:10.1186/1471-2105-9-523)
Supplement: Additional File 1 — A variety of html documents from the USeq web site detailing the available applications, their best usage, output file type descriptions, command line menus, etc. [file 1471-2105-9-523-S1.zip › USeqUserGuides/JavaDoc.zip!/JavaDoc/trans/misc/ConvertAgilentData.html]

ConvertAgilentData


|  |  |  |
| --- | --- | --- |
| **Overview** \| **Package** \| **Class** \| **Use** \| **Tree** \| **Deprecated** \| **Index** \| **Help** | |  |
| **PREV CLASS**   **NEXT CLASS** | **FRAMES**    **NO FRAMES**     **All Classes** |  |
| SUMMARY: NESTED \| FIELD \| CONSTR \| METHOD | DETAIL: FIELD \| CONSTR \| METHOD |  |


---


## trans.misc Class ConvertAgilentData

```
java.lang.Object
  trans.misc.ConvertAgilentData
```

---

``` public class ConvertAgilentData extends java.lang.Object ```

Reads in an Agilent tiling data file and outputs a tpmap and four cela files.
Cela files only contain intensities for chromosome mapped oligos, control intensities are set to zero.
Agilent uses zero based inter digit numbering, just like UCSC. Not necessarily! I've found end base included.
Assumes each oligo is a 60mer. Some arrays are isothermal so the actual oligo length varies.

---

**Constructor Summary**

`ConvertAgilentData(java.lang.String[] args)`


| **Method Summary** | |
| --- | --- |
| `static void` | `main(java.lang.String[] args)` |
| `boolean` | `parseAgilentFile()` |
| `static void` | `printDocs()` |
| `void` | `processArgs(java.lang.String[] args)`             This method will process each argument and assign new varibles |
| `boolean` | `save1lq()` |
| `boolean` | `saveTPMap()` |
| `void` | `writeCelaFiles()` |
| `boolean` | `writeSgrFiles()` |

**Methods inherited from class java.lang.Object**

`equals, getClass, hashCode, notify, notifyAll, toString, wait, wait, wait`

**Constructor Detail**

### ConvertAgilentData

```
public ConvertAgilentData(java.lang.String[] args)
```


**Method Detail**

### parseAgilentFile

```
public boolean parseAgilentFile()
```

---


### writeCelaFiles

```
public void writeCelaFiles()
```

---


### writeSgrFiles

```
public boolean writeSgrFiles()
```

---


### saveTPMap

```
public boolean saveTPMap()
```

---


### save1lq

```
public boolean save1lq()
```

---


### processArgs

```
public void processArgs(java.lang.String[] args)
```

:   This method will process each argument and assign new varibles

---


### printDocs

```
public static void printDocs()
```

---


### main

```
public static void main(java.lang.String[] args)
```


---


|  |  |  |
| --- | --- | --- |
| **Overview** \| **Package** \| **Class** \| **Use** \| **Tree** \| **Deprecated** \| **Index** \| **Help** | |  |
| **PREV CLASS**   **NEXT CLASS** | **FRAMES**    **NO FRAMES**     **All Classes** |  |
| SUMMARY: NESTED \| FIELD \| CONSTR \| METHOD | DETAIL: FIELD \| CONSTR \| METHOD |  |


---
